# Iron-Loaded Carbon Aerogels Derived from Bamboo Cellulose Fibers as Efficient Adsorbents for Cr(VI) Removal

**DOI:** 10.3390/polym13244338

**Published:** 2021-12-11

**Authors:** Xiaolin Xue, Wei Yuan, Zhuo Zheng, Jian Zhang, Chenghong Ao, Jiangqi Zhao, Qunhao Wang, Wei Zhang, Canhui Lu

**Affiliations:** 1 State Key Laboratory of Polymer Materials Engineering, Polymer Research Institute at Sichuan University, Chengdu 610065, China; xuexiaolin@stu.scu.edu.cn (X.X.); Union_sing@126.com (W.Y.); zhuo_zheng@scu.edu.cn (Z.Z.); pirlo3721@126.com (J.Z.); chenghongaocd@163.com (C.A.); scu_jqz@163.com (J.Z.); wangqunhao@stu.scu.edu.cn (Q.W.); 2 Advanced Polymer Materials Research Center of Sichuan University, Shishi 362700, China

**Keywords:** cellulose, iron, carbon aerogel, water treatment, adsorption

## Abstract

A unique iron/carbon aerogel (Fe/CA) was prepared via pyrolysis using ferric nitrate and bamboo cellulose fibers as the precursors, which could be used for high-efficiency removal of toxic Cr(VI) from wastewaters. Its composition and crystalline structures were characterized by FTIR, XPS, and XRD. In SEM images, the aerogel was highly porous with abundant interconnected pores, and its carbon-fiber skeleton was evenly covered by iron particles. Such structures greatly promoted both adsorption and redox reaction of Cr(VI) and endowed Fe/CA with a superb adsorption capacity of Cr(VI) (182 mg/g) with a fast adsorption rate (only 8 min to reach adsorption equilibrium), which outperformed many other adsorbents. Furthermore, the adsorption kinetics and isotherms were also investigated. The experiment data could be much better fitted by the pseudo-second-order kinetics model with a high correlating coefficient, suggesting that the Cr(VI) adsorption of Fe/CA was a chemical adsorption process. Meanwhile, the Langmuir model was found to better describe the isotherm curves, which implied the possible monolayer adsorption mechanism. It is noteworthy that the aerogel adsorbent as a bulk material could be easily separated from the water after adsorption, showing high potential in real-world water treatment.

## 1. Introduction

In modern society, heavy metal ions have become an emerging threat to the ecosystem because of high toxicity [1,2,3]. Among various heavy metals, hexavalent chromium (Cr(VI)) is classified as one of the most harmful pollutants in wastewater due to its acute toxicity to skin and living organisms and even potential carcinogenicity for human beings. The Cr(VI) usually exists in the form of soluble and mobile chromate ions (HCrO_4_^−^ or Cr_2_O_7_^2−^). It can migrate freely in the aqueous environment and is more likely to expand water pollution. Therefore, the removal of Cr(VI) from contaminated water before discharge is of critical importance for the safety of our ecosystem [4,5,6]. During the past few decades, scientists have attempted to develop a variety of strategies to remove Cr(VI) from wastewaters, such as membrane filtration, ozone treatment, ion exchange, chemical precipitation, physical/chemical adsorption, electron deposition, and photocatalyst [7,8,9,10,11]. Among them, adsorption is considered to be one of the most efficient and economical approaches without any by-products and secondary pollution [12,13,14]. Thus far, numerous adsorbents have been applied to remove Cr(VI) in wastewater, such as phosphates, clay minerals, zeolites, and carbon materials [15,16,17,18]. In particular, owing to the highly porous structure and tunable chemical properties, carbon materials are regarded as promising sorbents for wastewater treatment [19].

In general, the removal process for Cr(VI) by carbon materials can be divided into two steps: adsorption and redox reaction. Firstly, the Cr(VI) ions diffuse into the adsorbents and then are adsorbed thereof at the surface of adsorbents by various interactions. Secondly, the adsorbed Cr(VI) ions react with the reductive groups on the adsorbents and ultimately are reduced to Cr(Ⅲ) [20]. Notably, Cr(Ⅲ) has low toxicity and actually is an essential nutrient to metastasize sugar and fat in the human body. Fe^0^ and Fe^2+^ are often used to promote the reduction of Cr(VI) (Equation (1)) for their high affinity and selectivity toward Cr(VI) [21], whereas the surface functional groups on carbon materials (OH, COOH, C=O, etc.), coupled with the abundant sp2 carbon, favor the dispersion of iron and iron oxide in the carbon matrix and also help reduce Cr(VI) to Cr(Ⅲ). Consequently, considerable research efforts have been focused on loading Fe^0^ onto carbon materials with ambitions to develop next generation Cr(VI) adsorbents that combine the merits of carbon materials and Fe^0^ [12,22,23].

2CrO_4_^2−^ + 3Fe^0^ + 16H^+^ → 2Cr^3+^ + 3Fe^2+^ + 8H_2_O (1)

Compared with the expensive carbon materials such as carbon nanotubes and graphene, the ones from biomass precursors show distinctive advantages for water treatment. They are low in cost with sustainable and renewable supplies but also more effective for the adsorption of Cr(VI) as bearing a large amount of oxygen-containing groups [12,24]. Dong et al. [25] used cornstalk and ferric chloride as raw materials to prepare nanoscale zero-valent iron/biochar composites. The adsorption capacity for Cr(VI) could reach 12 mg/g. Han et al. [26] reported magnetic biochar prepared from peanut hull and ferric chloride through pyrolysis, and its adsorption capacity of Cr(VI) was 77 mg/g. Zhuang et al. [27] prepared a carbon composite by reducing Fe(NO_3_)_3_ with starch as a matrix, and the Cr(VI) adsorption capacity was 10 mg/g.

Although these early investigations have demonstrated promising potentials of biomass-derived carbons for Cr(VI) remediation, their performances are still far from expectations. This is mainly ascribed to the limited or separated pore structures, which suppress the diffusion of Cr(VI) to approach the active sites on carbons, leading to a poor adsorption capacity. Additionally, most of them are presented in powder form. Therefore, some energy-intensive post-processes to separate adsorbents from water, such as centrifuging, are required after adsorption completion, which further hinders their industrial applications in water treatment. Thus, the development of an adsorbent with the desired characteristics of high adsorption efficiency and facile post-separation remains a challenge.

Carbon aerogel, a new species of carbon material, is usually characterized as having an ultra-low density and a large specific surface area with numerous interconnected pores, which are favorable for the adsorption of heavy metal ions. In this study, a novel carbon aerogel, Fe/CA, derived from bamboo cellulose with high efficiency for Cr(VI) removal, was fabricated by loading zero-valent iron homogenously on the carbon aerogel [28]. The abundant interconnected pores inside the aerogel provided adequate spaces to accommodate heavy metal ions and promoted the diffusion of these ions for rapid removal. In addition, the synergistic effect of multi-reductive sites (a large number of oxygen-containing groups, Fe^0^ and Fe^2+^) could also benefit the reduction process of Cr(VI). As a result, the two key processes for Cr(VI) removal (adsorption and redox reaction) would be simultaneously enhanced, leading to superb adsorption performance. The effects of pH, initial Cr(VI) concentration, and contact time on the adsorption capacity were systematically investigated. In addition, the Cr(VI) removal kinetics and isotherms were also studied. 

## 2. Materials and Methods

### 2.1. Materials

Bamboo pulp was supplied by Yongfeng Paper Co., Ltd. (Muchuan, China). Analytically pure Fe(NO_3_)_3_·9H_2_O and potassium dichromate (K_2_Cr_2_O_7_) were obtained from Kelong Chemical Reagent Co., Ltd. (Chengdu, China). 

### 2.2. Synthesis of Fe/CA

**Figure 1:**
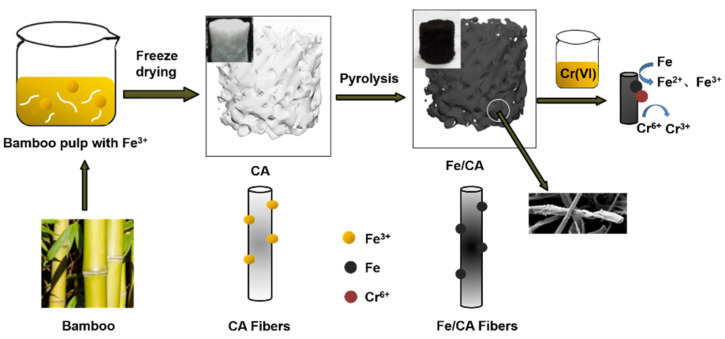
Schematic of the synthesis of Fe/CA.

Figure 1 shows the schematic of the synthesis process of Fe/CA. First, the bamboo pulp was uniformly dispersed into deionized water under vigorous magnetic stirring to form a homogenous suspension with a concentration of 1 wt.%. Then, Fe(NO_3_)_3_·9H_2_O with an equal mass of cellulose was added into the above suspension and further stirred for 3 h at room temperature. After that, the suspension was freeze-dried at −40 °C for 48 h to obtain the Fe(NO_3_)_3_/cellulose hybrid aerogel.

Next, the obtained hybrid aerogel was placed in a tube furnace (OTF1200X, Kejing Materials Technology Co., Ltd., Hefei, China) for pyrolysis. The pyrolysis process included five main steps. First, the temperature was increased to 240 °C at a rate of 2 °C min^−1^ and stayed at this temperature for 1 h. Then, the temperature was increased to 400 °C at a heating rate of 2 °C min^−1^ and kept at this temperature for 1 h. After that, the temperature was further increased to 800 °C at a rate of 5 °C min^−1^ and kept at this temperature for another 2 h. Finally, the temperature was decreased to 400 °C within 80 min and then naturally decreased to room temperature. All processes were carried out under a nitrogen atmosphere. For comparison, an aerogel from neat bamboo cellulose fibers without Fe(NO_3_)_3_·9H_2_O was treated in the same way as described above, and the product was denoted as CA.

### 2.3. Characterization 

The micromorphology of Fe/CA was observed by the field-emission scanning electron microscopy (FESEM, JEOL JSM-7500F, Tokyo, Japan) at an accelerating voltage of 20.0 kV. The relative contents of the elements were analyzed via the energy-dispersive X-ray spectroscopy (EDS). The fiber diameters were measured from the SEM images by Image J software. The chemical structures of Fe/CA were characterized by Fourier transform infrared spectroscopy (FTIR, NICOLET 6700, Thermo Fisher Scientific, Waltham, MA, USA) in the range of 400–4000 cm^−1^ with a resolution of 4 cm^−1^. The chemical composition of Fe/CA and the form of Cr ions were characterized by X-ray photoelectron spectroscopy (XPS, ESCALAB 250Xi, Thermo Scientific, Waltham, MA, USA) with an Al Kα X-ray source (1486.8 eV) and an X-ray beam of around 0.5 mm. The specific surface area of Fe/CA was determined by nitrogen adsorption–desorption experiments at −196 °C, using an accelerated surface area and porosimetry system (ASAP2020, Micromeritics, Norcross, GA, USA). The crystal structure of iron was disclosed by the powder X-ray diffraction (XRD, D8 Advance, Bruker, Karlsruhe, Germany) pattern with Cu Kα radiation at a wavelength (λ) of 1.541 Å. The Raman spectra were obtained on Raman Microspectrometer (STA 6000, PerkinElmer, Waltham, MA, USA), with an excitation wavelength of 532 nm.

### 2.4. Batch Adsorption Experiments

#### 2.4.1. The Effect of pH on Cr(VI) Removal Efficiency 

A total of 20 mg of Fe/CA was immersed into 50 mL of K_2_Cr_2_O_7_ solution (20 mg/L). The pH range of the solution was 3.0–8.0 and was adjusted by NaOH (1 M) and H_2_SO_4_ (1 M). Then, the solution was shaken at room temperature in a water bath shaker (HY-3A, Changzhou Henglong Instrument Co., Ltd. Changzhou, China). When the adsorption reached equilibrium, the remaining Cr(VI) concentration was measured by the colorimetric method [29]. The relationship between Cr(VI) concentration and light absorbance was governed by Equation (2), and the adsorption capacity for Cr(VI) was calculated by Equation (3).

*C* = 0.63419*A* + 0.01153 (2)

where *C* is the remaining concentration of Cr(VI); *A* is the absorbance at 540 nm detected by the UV–visible spectrophotometer (UV-1800, Mapada Instruments Co., Shanghai, China).

(3) $$q_{e}=\frac{\left(C_{0}-C_{e}\right)V}{m}$$

where *q_e_* (mg/g) is the adsorption capacity; *C_0_* and *C_e_* (mg/L) are the concentrations of Cr(VI) before and after treatment, respectively; *m* (mg) is the mass of Fe/CA; *V* (mL) is the volume of the Cr(VI) solution.

#### 2.4.2. The Effect of Initial Cr(VI) Concentration on Cr(Ⅵ) Removal Efficiency 

A total of 20 mg of Fe/CA was immersed into a series of 50 mL K_2_Cr_2_O_7_ solutions with concentrations in the range of 20–500 mg/L. The pH of the solution was fixed at 3. Then, the solution was shaken at room temperature in a water bath shaker. Additionally, the adsorption capacity was obtained by the same method described above.

#### 2.4.3. The Effect of Contact Time on Cr(VI) Removal Efficiency

In a typical process, 18 mg of Fe/CA was immersed into a K_2_Cr_2_O_7_ solution (50 mL, 200 mg/L). The pH of the solution was fixed at 3. Then, the solution was shaken at room temperature in a water bath shaker. At the preset interval, the concentrations of Cr(VI) that remained in the solution were measured, and the adsorption capacity at time t was calculated by Equation (4).

(4) $$q_{t}=\frac{\left(C_{0}-C_{t}\right)V}{m}$$

where *q_t_* (mg/g) is the adsorption capacity at time *t*; *C*_0_ and *C_t_* (mg/L) are initial and time *t* Cr(VI) concentrations, respectively; *m* (mg) is the mass of Fe/CA; *V* (mL) is the volume of the Cr(VI) solution.

## 3. Results and Discussion

### 3.1. The Morphological and Structural Characterizations of CA and Fe/CA

**Figure 2:**
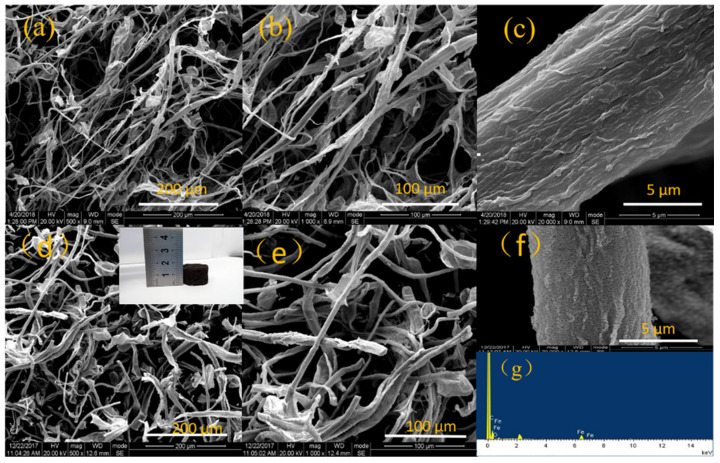
SEM images of the fractured surface of CA (**a**–**c**) and Fe/CA (**d**–**f**). EDS spectrum of Fe/CA (**g**). A photograph of Fe/CA is inserted in (**d**).

Figure 2 displays the SEM images of CA and Fe/CA. Additionally, a digital photo image of the obtained Fe/CA is presented in Figure 2d, showing its three-dimensional geometry with black color. Compared with CA, the average diameter of carbonized fibers in Fe/CA increased from 11 μm to 13 μm. The Fe peak was found in the EDS spectrum of Fe/CA (Figure 2g), which confirmed the anchoring of Fe onto the carbon skeleton. The surface of Fe/CA became rougher than that of CA. From Figure 2d–f, it was observed that iron was well distributed along the fibers and formed an iron layer on their surface. This structure could increase the number of reductive sites and promote the reduction reaction of Cr(VI), giving rise to improved adsorption capacity. Importantly, Fe/CA still retained the highly porous structure of CA. The high porosity of Fe/CA was favorable for Cr(VI) ions removal in different aspects. On the one hand, the abundant interconnected pores of Fe/CA with a large specific surface provide more adsorption sites for Cr(VI) because adsorption mainly occurs at the material’s surface, and they also offer numerous channels for rapid diffusion of Cr(VI) inside the materials, which effectively promoted the first adsorption process. On the other hand, the porous structure also facilitates a higher loading of active species such as Fe^0^, which induces a more effective reduction of Cr(VI) to Cr(III). As a consequence, the whole removal efficacy of Cr(VI) could be greatly improved [23].

**Figure 3:**
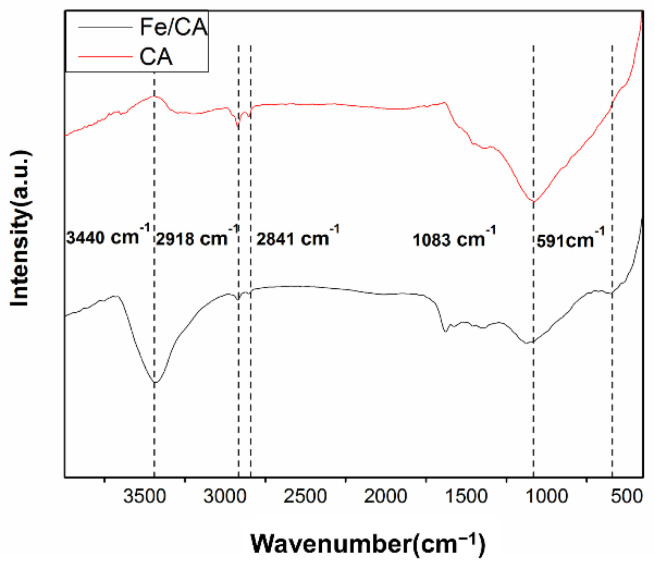
FTIR spectra of CA and Fe/CA.

Figure 3 displays the FTIR spectra of Fe/CA and CA. For CA, the bands at 2920–2850 cm^−1^ were assigned to the vibration of CH and CH_2_. Additionally, the peak appeared at 1083 cm^−1^ corresponded to C–O. After comparing the two FTIR spectra, the most prominent difference of peak intensity was found for the peak at 3440 cm^−1^ assigned to the –OH groups. The –OH absorbance in CA almost completely diminished after the pyrolysis process. However, the Fe/CA still displayed a distinct –OH peak. This is normal and well in line with some similar systems reported in the literature [25,30]. When loaded on the biomass, the Fe^3+^ ions were firstly hydrolyzed to form Fe hydroxides (FeO(OH)). During the next pyrolysis process, the iron-preloaded biomass was thermally decomposed, accompanied by the reduction of Fe^3+^ to Fe^0^. Nonetheless, a part of Fe hydroxides (FeO(OH)) could still be retained in the resultant Fe/CA [31]. Notably, these –OH groups would be conducive to the homogeneous distribution of iron on the carbon fibers, offering abundant active sites for improving adsorption capacity [22,32]. Moreover, the band at 591 cm^−1^ for Fe/CA represented the stretching vibration of Fe–O, which implied that those surface zero-valent irons were partially oxidized [33].

**Figure 4:**
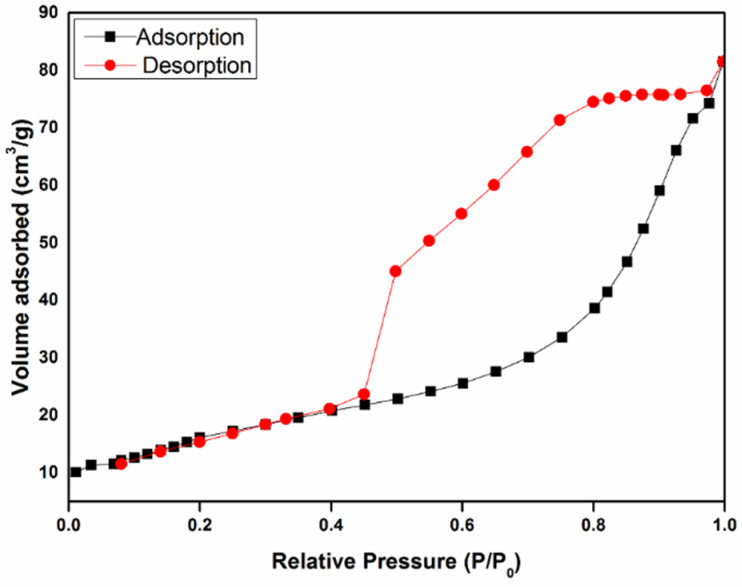
N_2_ adsorption–desorption isotherms of Fe/CA.

The obtained Fe/CA displayed an ultra-low density of 13.5 mg/cm^3^. In addition, the specific surface area of Fe/CA was determined by nitrogen adsorption–desorption isotherms, and the results are shown in Figure 4. The Fe/CA exhibited type-IV adsorption isotherm according to the IUPAC classification. Additionally, an obvious hysteresis loop could be observed at higher N_2_ pressure (P/P_0_ = 0.42−0.99), suggesting the dominated existence of micropores and mesopores. This hysteresis loop was usually related to the capillary condensation from the interconnected pore system. The specific surface area and pore volume of Fe/CA were 135.4 m^2^/g and 0.13 cm^3^/g, respectively. Compared with CA [28], both the specific surface area and pore volume of Fe/CA decreased due to the fact that the loaded irons occupied some micropores of CA. Nonetheless, the specific surface area of Fe/CA was still higher than those of many other materials of this kind, such as the zero-valent iron/biochar activated by KOH or HCl (5.4–39 m^2^/g) [25], the herb-based biochar/iron composite (59.3 m^2^/g) [34], and the tea waste/iron porous carbonaceous material (31 m^2^/g) [35].

**Figure 5:**
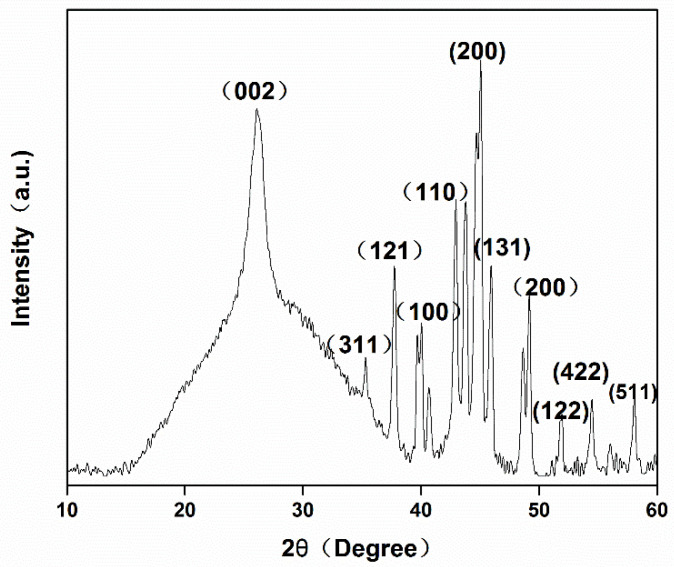
XRD pattern of Fe/CA.

The crystalline structure was characterized by XRD. Figure 5 displays the XRD pattern of Fe/CA. The diffraction at 26.09° was assigned to the graphite plane (002), suggesting the presence of graphite-like materials. The peaks at 40.2° and 42.9° corresponded to the (100), (110) planes of FeO and α-Fe, respectively [36], whereas the peaks at 45.0° and 49.4° corresponded to the (200) planes of γ-Fe [37]. In addition, the diffractions at 37.7°, 48.6°, and 51.8° represented the (121), (131), and (122) planes of Fe_3_C, respectively, which manifested that some iron had reacted with carbon to form Fe–C. This structure would effectively prevent the aggregation of iron and promote the interaction between iron and Cr(VI) [38]. In addition, the weak diffractions at 35.5° and 56.9° assigned to (311) and (511) planes of Fe_3_O_4_ indicated that a small part of zero-valent iron had been oxidized during carbonization. However, the high-intensity ratio of Fe to Fe_3_O_4_ implied that most of the iron was still in the form of zero-valent iron.

**Figure 6:**
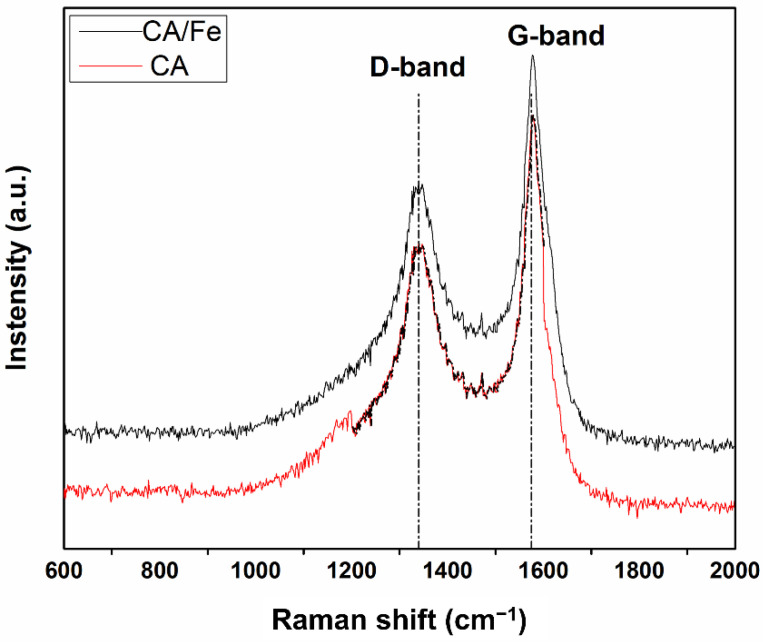
Raman spectra of Fe/CA and CA.

Figure 6 depicts the Raman spectra of the samples. The bands located at 1336 cm^−1^ and 1588 cm^−1^ corresponded to the D and G bands of C–C and C=C stretching vibration, respectively. The intensity ratio of the D band and G band (I_D_/I_G_) could be used to estimate the disorder degree of carbon materials. The I_D_/I_G_ of Fe/CA was calculated to be 0.74, higher than that of CA (0.69), indicating that the material was mainly in the form of amorphous carbon and the addition of iron produced more defects in the composite with a decrease in in-plane graphitic crystallites. This result could be attributed to the formation of Fe_3_C during the carbonization process, which disrupted the C-C ordering in the graphite skeleton [39].

**Figure 7:**
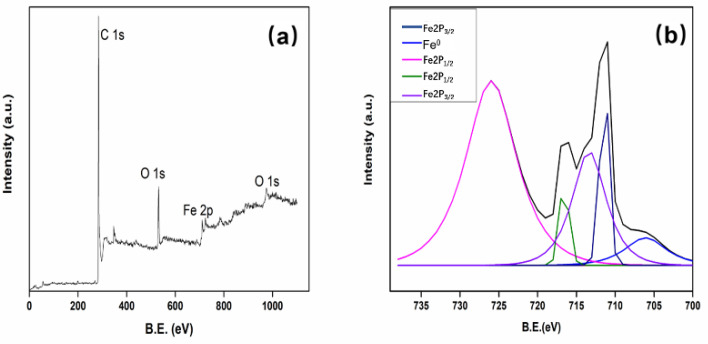
XPS spectrum (**a**) and deconvoluted Fe 2p spectrum (**b**) of Fe/CA.

XPS was commonly applied to characterize the chemical composition of carbon materials. As seen in Figure 7, three elements—Fe, C, and O were found in the XPS survey spectrum of Fe/CA, suggesting that iron had been incorporated into the carbon matrix successfully. The high-resolution spectrum of Fe2p is shown in Figure 7b. The peaks positions located at 711.6 eV, 713.3 eV, 717.2 eV, and 726.3 eV were assigned to Fe2p_3/2_ and Fe2p_1/2_, respectively. These feature peaks indicated that the surface of Fe/CA might contain a layer of iron oxides, likely in the form of FeO, Fe_2_O_3_, and Fe_3_O_4_ [40]. The small shoulder at around 706.1 eV was for the 2p_3/2_ peak of zero-valent iron (Fe^0^) [41]. However, according to the XRD pattern, most irons were presented in the form of Fe^0^. Such seemingly contradictory results could be explained by the difference in the detecting principles between the two techniques. Generally, XPS characterizes the chemical composition of a substance in about several hundred nanometers in thickness [42]. No wonder that the surface compositions of Fe/CA revealed by XPS mainly comprised iron oxide, as the iron loaded on the fiber surface could be easily oxidized in ambient conditions. By contrast, XRD analyzes the overall crystalline structure of materials. Hence, a higher Fe^0^ content was recorded from XRD data.

### 3.2. Cr(VI) Removal Performance

#### 3.2.1. The Effect of pH on the Adsorption Capacity

**Figure 8:**
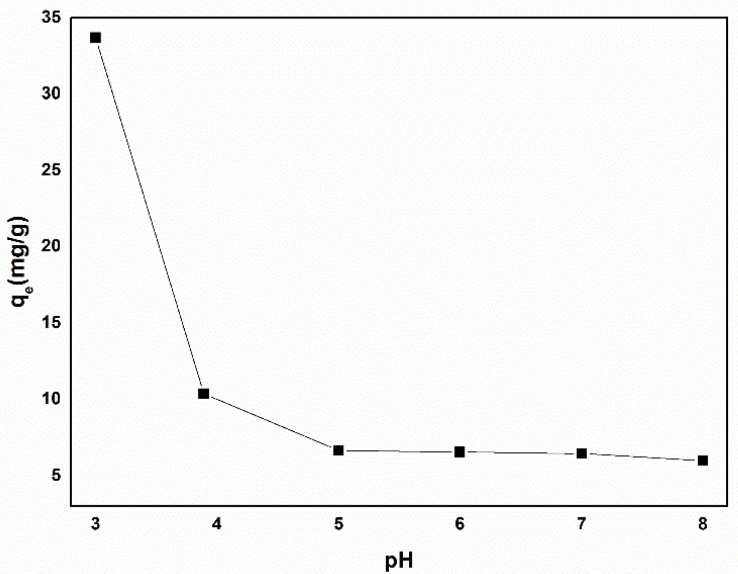
The effect of pH on the Cr(VI) adsorption capacity of Fe/CA.

The pH of the solution played a crucial role in the adsorption process. In this study, 20 mg of Fe/CA was applied to the K_2_Cr_2_O_4_ solutions (20 mg/L), with pH ranging from 3.0 to 8.0. Figure 8 shows the adsorption capacity for Cr(VI) under different pH values. The maximum adsorption capacity was 35 mg/g at pH = 3.0. However, with the increase in pH, the adsorption capacity of Fe/CA decreased significantly, and the lowest adsorption capacity was only 5 mg/g. As is well recognized, Cr(VI) exists in different forms at different pH, including CrO_4_^2−^, HCrO_4_^−^, H_2_CrO_4_, Cr_2_O_7_^2−^ and HCr_2_O_7_^−^. Additionally, there are four equilibrium reactions among these five Cr(VI) species [43,44] as follows:

CrO_4_^2−^ + H^+^ ⇌ HCrO_4_^−^ (5)

HCrO_4_^−^ + H^+^ ⇌ H_2_CrO_4_ (6)

2HCrO_4_^−^ ⇌ Cr_2_O_7_^2−^ + H_2_O (7)

Cr_2_O_7_^2−^ + H^+^ ⇌ HCr_2_O_7_^−^ (8)

When pH was lower than 6.8, the Cr(VI) ions mainly existed in the forms of Cr_2_O_7_^2−^, HCrO_4_^−^ and HCr_2_O_7_^−^, whereas CrO_4_^2−^ would become the dominating species when pH was above 6.8 [23,45]. In an acidic solution, the presence of H^+^ could enhance the oxidability of Cr_2_O_7_^2−^ and HCrO_4_^−^, promoting the chemical reaction between Cr(VI) ions and FeO, α-Fe, and γ-Fe, consequently resulting in a higher adsorption capacity for Cr(VI). Moreover, the surface chemistry of material also played an important role in the whole adsorption process. As reported by Oliveira et al. [46], the pH at zero charge (pH_pzc_) of biomass-based carbon material was about 4.5. When pH was lower than pH_pzc_, the surface of materials was positively charged, as the group X–OH (X = Fe, C) in Fe/CA would form X–OH_2_^+^. Therefore, the negatively charged Cr(VI) could be easily attracted by Fe/CA via electrostatic interactions. However, when pH was above pH_pzc_, the surface of materials became negatively charged, leading to poor Cr(VI) adsorption. It is also important to note that the concentration of OH^−^ increased with the increase in pH, which induced adsorption competition between OH^−^ and Cr(VI) and further deteriorated the adsorption capacity.

#### 3.2.2. Adsorption Isotherms

**Figure 9:**
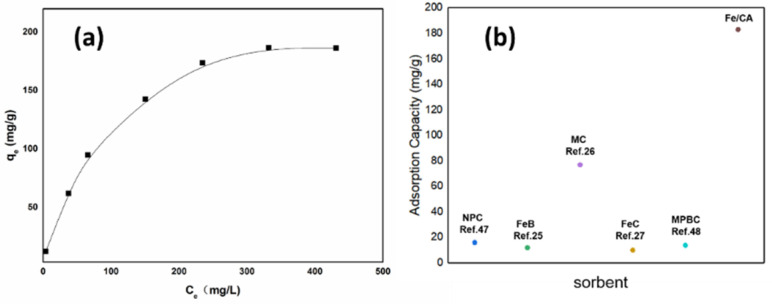
The adsorption isotherm (**a**) of Fe/CA and the comparison of Cr (VI) adsorption capacities for different adsorbents (**b**).

Figure 9a depicts the adsorption performance under different initial Cr(VI) concentrations with a fixed adsorbent dosage of 0.3 g/L. Generally, the Cr(VI) adsorption capacity of Fe/CA increased with the increase in the initial Cr(VI) concentration. The maximum adsorption capacity could reach 182 mg/g at an initial Cr(VI) concentration of 400 mg/L. However, further increment of the initial Cr(VI) concentration did not bring about a higher adsorption capacity to the adsorbent possibly due to the fact that its active sites had been fully occupied by the adsorbed heavy metal ions. Figure 9b compares the adsorption capacities of Fe/CA and some other adsorbents reported in the literature. Clearly, Fe/CA presented a superior adsorption capacity in comparison with other ones, such as N-doped porous carbon with magnetic particles (NPC, 16mg/g) [47], nanoscale zero-valent iron/biochar composites (FeB, 12 mg/g) [25], magnetic biochar (MC, 77 mg/g) prepared from peanut hull and ferric chloride [26], carbon-encapsulated iron composite (FeC, 10 mg/g) [27], and magnetic biochar from pine sawdust (MPBC, 13.83 mg/g) [48].

Furthermore, the Langmuir and Freundlich adsorption isotherm models were used to fit the experimental data of Cr(VI) adsorption, respectively. Additionally, the Langmuir isotherm is described as follows:

(9) $$\frac{C_{e}}{q_{e}}=\frac{1}{bq_{m}}+\frac{c_{e}}{q_{m}}$$

where *C_e_* (mg/L) is the equilibrium concentration of Cr(VI); *q_e_* (mg/g) is the adsorption capacity of Cr(VI) at equilibrium; *q_m_* (mg/g) is the adsorption capacity of adsorbents; *b* (L/mg) is a constant.

Freundlich isotherm is an empirical model, which values the heterogeneous adsorptive energies on the surface of the absorbent and can be depicted as follows:

(10) $$\log\;q_{e}=\log\;{k_{f}}_{\;}+\frac{1}{n\;}\;\log\;C_{e}$$

where *q_e_* (mg/g) is adsorption capacity at equilibrium; *k_f_* and n are constants of the model; *C_e_* (mg/L) is the equilibrium concentration of Cr(VI).

**Figure 10:**
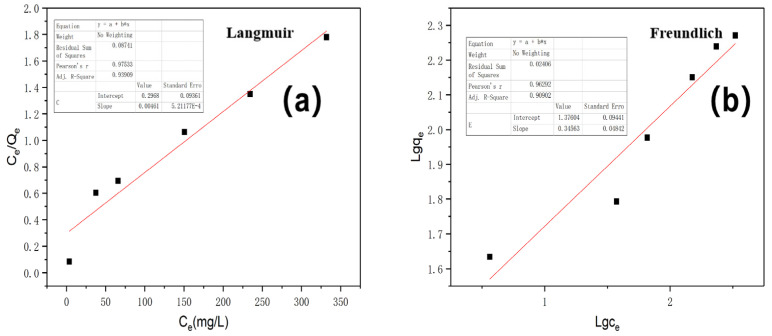
The Cr(VI) adsorption isotherm fitted by (**a**) Langmuir and (**b**) Freundlich models.

The adsorption isotherms fitted by both Langmuir and Freundlich models are depicted in Figure 10. The correlation coefficient (R^2^) value of the Langmuir model (0.93909) was higher than that of the Freundlich model (0.90902), indicating that the Cr(VI) adsorption behavior of Fe/CA could be better described by the Langmuir model and that it was a monolayer adsorption process [49].

#### 3.2.3. Adsorption Kinetics

**Figure 11:**
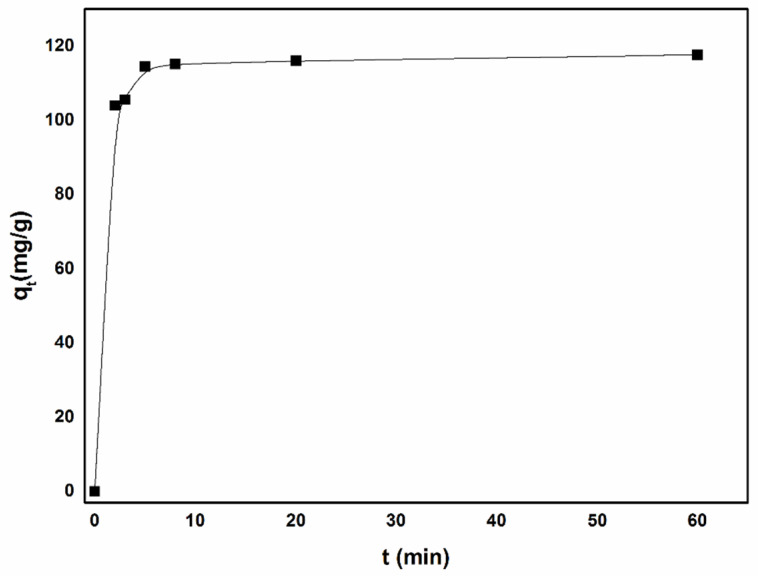
The Cr(VI) adsorption kinetics of Fe/CA.

Figure 11 shows the effect of contact time on the adsorption capacity of Fe/CA in batch experiments. In the initial 3 min, the adsorption rapidly reached 80% of the maximum capacity, and the adsorption equilibrium could be achieved after 8 min. Zhang et al. [50] reported similar kinetics for Cr(VI) adsorption. Additionally, it could be attributed to the abundant interconnected pores inside Fe/CA, which provided massive passageways for the diffusion of Cr(VI). As a result, more Cr(VI) ions could diffuse into the interior of Fe/CA and interact with those surface-active species, leading to fast and effective adsorption of Cr(VI). In order to better interpret the adsorption behavior of Fe/CA, the pseudo-first-order (Equation (11)) and pseudo-second-order (Equation (12)) kinetics models were used to fit the experimental data, respectively.

(11) $$\mathrm{Ln}(q_{e}-q_{t})=\ln q_{e}-\frac{k_{1}}{2.0303}\;t$$

(12) $$\frac{t}{q_{t}}=\frac{1}{k_{2\;}q_{e}^{2}}+\frac{t}{q_{e}}$$

where *t* (min) is the adsorption time; *k*_1_ (min^−1^) and *k*_2_ (g/(mg/min)) are the constants about the pseudo-first-order and pseudo-second-order reaction rate equation, respectively. Additionally, *q_e_* (mg/g) is the adsorption capacity at equilibrium, and *q_t_* (mg/g) is the adsorption capacity at time *t*.

**Figure 12:**
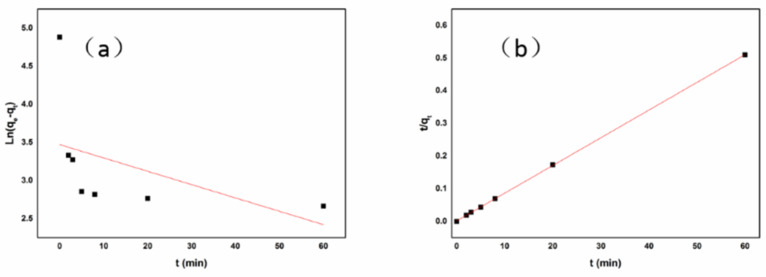
The Cr(VI) adsorption kinetics fitted by the pseudo-first-order (**a**) and pseudo-second-order (**b**) models.

Figure 12 shows the adsorption kinetics of Cr(VI). The correlation coefficient (R^2^) value of the pseudo-second-order model was 0.99995, much higher than that of the pseudo-first-order model (0.08000). This result strongly indicated that the adsorption of Cr(VI) by Fe/CA was governed by the pseudo-second-order model, and the adsorption was a diffusion-controlled process [26].

### 3.3. Cr(VI) Removal Mechanism

**Figure 13:**
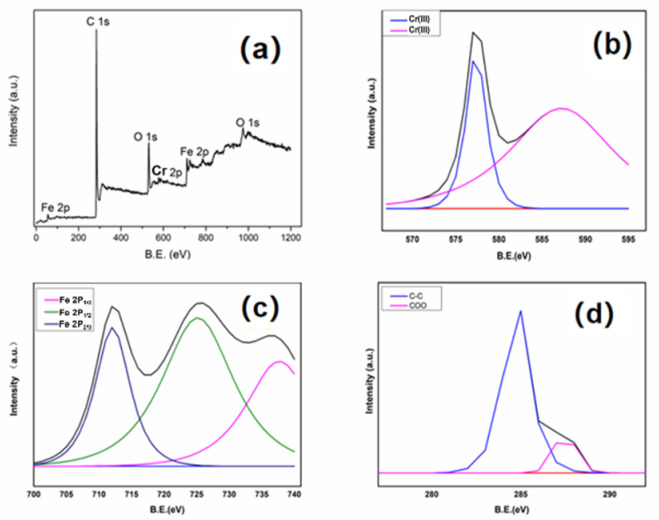
The XPS spectrum of Fe/CA after treating with Cr(VI) (**a**) and the corresponding deconvoluted Cr2p (**b**), Fe2p (**c**), and C1s (**d**) spectra of Fe/CA.

In order to reveal the Cr(VI) adsorption mechanism, XPS was used to characterize the surface chemical composition of Fe/CA after service, and the results are shown in Figure 13. There were only four elements—Fe, Cr, C, and O in the XPS survey spectrum of Fe/CA (Figure 13a), which manifested that the Cr(VI) had indeed been adsorbed on Fe/CA. Figure 13b shows the Cr2p spectrum. The bands at BE = 576.85 and 587.3 eV confirmed that the adsorbed Cr ions on Fe/CA were in the form of Cr(Ⅲ). Figure 13c displays the Fe2p spectrum of Fe/CA after treating with Cr(VI). The peak centered at 706.1 eV for Fe^0^ disappeared, suggesting that Fe^0^ had been actively involved in the reduction of Cr(VI) during the adsorption process [44,45]. Figure 13d is the C1s spectrum of Fe/CA after adsorption. The peaks at 284.9 eV and 287.6 eV were assigned to C–C and COO, respectively. This result indicated that the carbon atoms might also participate in the reduction of Cr(VI) to Cr(Ⅲ), as described in Equation (13).

HCrO_4_^−^ (aq) + carbons(s) → Cr^3+^ (aq) + carbons O(s) (13)

Moreover, some reductive intermediates generated during the adsorption process, such as H radical (H*) and Fe^2+^, would also act as electron donors to promote the reduction of Cr(VI). According to the literature [49], after the adsorption process in an acid solution, the pH of the solution would increase, which meant the H^+^ could be consumed during the treatment. In an acidic solution, partial Fe^0^ loaded on the adsorbent would react with H^+^ to produce Fe^2+^, which was accompanied by an increase in pH. Meanwhile, some intermediates such as electron (e^−^) and H radical (H*) were produced during this process, which might be responsible for the reduction of Cr(VI). Such a Cr(VI) removal process was described as follows:

Fe + H^+^ → Fe^2+^ + R (R = e^−^/H*) (14)

Cr(Ⅵ) + R → Cr(Ⅲ)(15)

Cr(Ⅵ) + Fe^2+^ → Cr(Ⅲ) + Fe^3+^ (16)

In neutral and alkaline solutions, the Cr_2_O_7_^2−^ in water could be reduced by Fe^0^, and the generated Cr(Ⅲ) would react with OH^−^ to form hydroxides. Specifically, one mol of Cr(VI) reacts with Fe to produce 2 mol Cr(Ⅲ), 2 mol Fe^3+^, and 14 mol OH^−^ (Equation (17)). However, only 12 mol OH^−^ were required to react with the Cr(Ⅲ) and Fe^3+^ (Equations (18) and (19)). Thus, it would cause the accumulation of OH^−^ and, consequently, a slight increase in pH, which well agreed with previous reports [49].

Cr_2_O_7_^2−^ + 2Fe + 7H_2_O → 2Cr^3+^ + 2Fe^3+^ + 14OH^−^ (17)

2Cr^3+^ + 6OH^−^ → 2Cr(OH)_3_ (18)

2Fe^3+^ + 6OH^−^ → 2Fe(OH)_3_ (19)

## 4. Conclusions

In this work, Fe/CA was prepared from cellulose and ferric nitrate via pyrolysis, which could be used as an effective adsorbent for Cr(VI) removal. In Fe/CA, the iron was homogenously loaded on the surface of carbonized fibers and presented mostly in the form of zero-valent iron. The obtained Fe/CA well preserved the highly porous structure of CA and exhibited an ultra-low density of 13.5 mg/cm^3^. The abundant interconnected pores of the adsorbent provided massive passageways for ion diffusion, which endowed it a superior adsorption capacity of Cr(VI) (182 mg/g) as well as a high adsorption rate (only 8 min to reach adsorption equilibrium). Furthermore, XPS studies indicated that most adsorbed Cr(VI) ions had been reduced to Cr(III) by Fe and carbon in the aerogel. It is envisaged that this high-performance Fe/CA from sustainable bioresources would serve as a promising adsorbent for Cr(VI) removal in future wastewater treatments.

## Data Availability

The data presented in this study are available within this article. Further inquiries may be directed to the authors.
